# Supplementary material for: Synthesis of Wrinkle-Free Metallic Thin Films in Polymer by Interfacial Instability Suppression with Nanoparticles
Source: Nanomaterials (Basel). 2023 Mar 14;13(6):1044. doi: 10.3390/nano13061044 (PMC10054355; doi:10.3390/nano13061044)
Supplement: Supplementary file 1 [file nanomaterials-13-01044-s001.zip › nanomaterials-2207779-supplementary.zip!/nanomaterials-2207779-supplementary.pdf]

nanomaterials

Supplementary Materials

Synthesis of Wrinkle-Free Metallic Thin Films in Polymer by Interfacial Instability Suppression with Nanoparticles

Maryam Jalali-Mousavi, Samuel Kok Suen Cheng and Jian Sheng *

College of Engineering, Texas A&M University–Corpus Christi, Corpus Christi, Texas 78412, USA; maryam.jalali-mousavi@tamucc.edu (M.J.-M.); koksuen.cheng@tamucc.edu (S.K.S.C.)
* Correspondence: jian.sheng@tamucc.edu; Tel.: +1-361-825-3731

Figure legend

Figure S1. Interfacial structures of a 500nm thick parylene-C jammer layer. (A) Sample micrographs of a 500nm thick parylene-C layer decorated with many nanoparticle-like aggregates. (B) 3D surface roughness over a sample area of $50 \times 50\, \mu m^2$. (C) Contour plot of (B). (D) 3D surface roughness over the second sample area. Both show many micron and nanoscale particle-like aggregates. (E) surface roughness of the substrate after removing jammer layer.

Figure S2. Spectra of WiMTiP samples with jammer thickness, t, of (**A**) 0, (**B**) 74, (**C**) 118 (**D**) 160um, (**E**) 340nm.

Figure S3. Sample $F - \delta$ curves of nano-indentation over an elastomer with a jammer layer of 0, 40, 130, 340nm thickness. Color coded by thickness

Figure S4. Hertian elasticity, E_0, vs the jammer thickness (nm)

Movie legend

Video S1. Microscopic video of the hydration of a dried agar film over a WiMTiP composite. The clip is played at 2X normal speed.

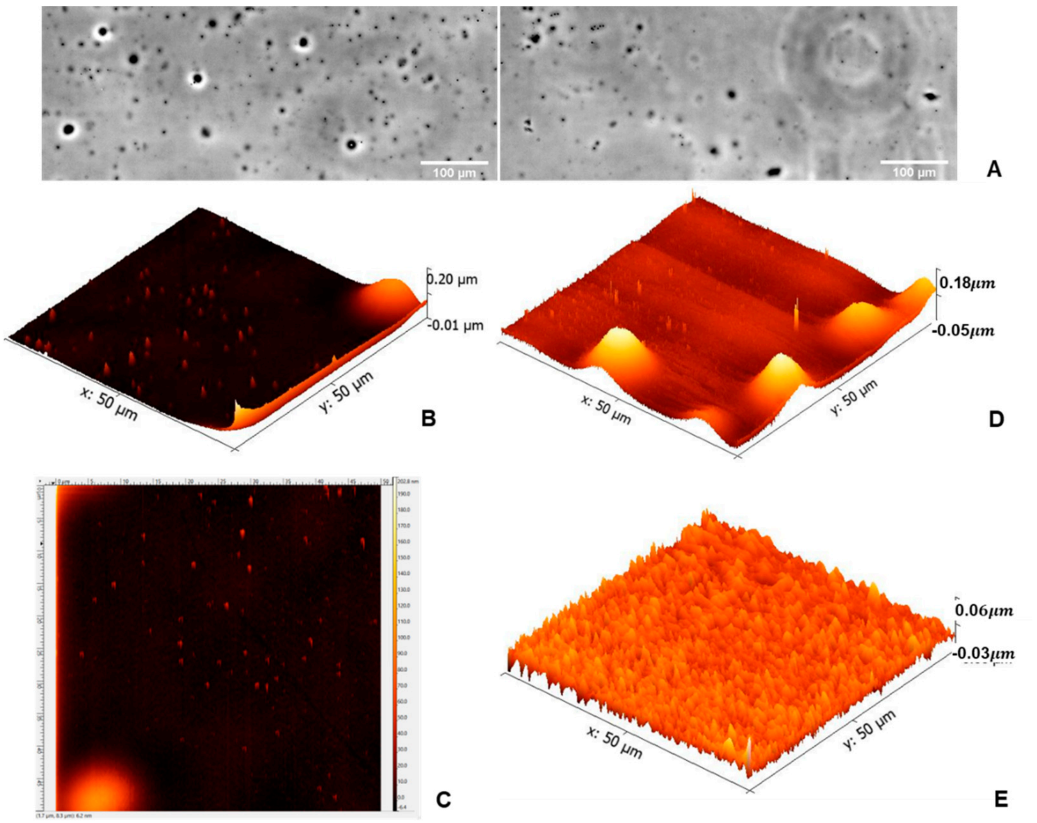

Figure S1. Interfacial structures of a 500nm thick parylene-C jammer layer. (A) Sample micrographs of a 500nm thick parylene-C layer decorated with many nanoparticle-like aggregates. (B) 3D surface roughness over a sample area of $50 \times 50\ \mu m^2$. (C) Contour plot of (B). (D) 3D surface roughness over the second sample area. Both show many micron and nanoscale particle-like aggregates. (E) surface roughness of the substrate after removing jammer layer.

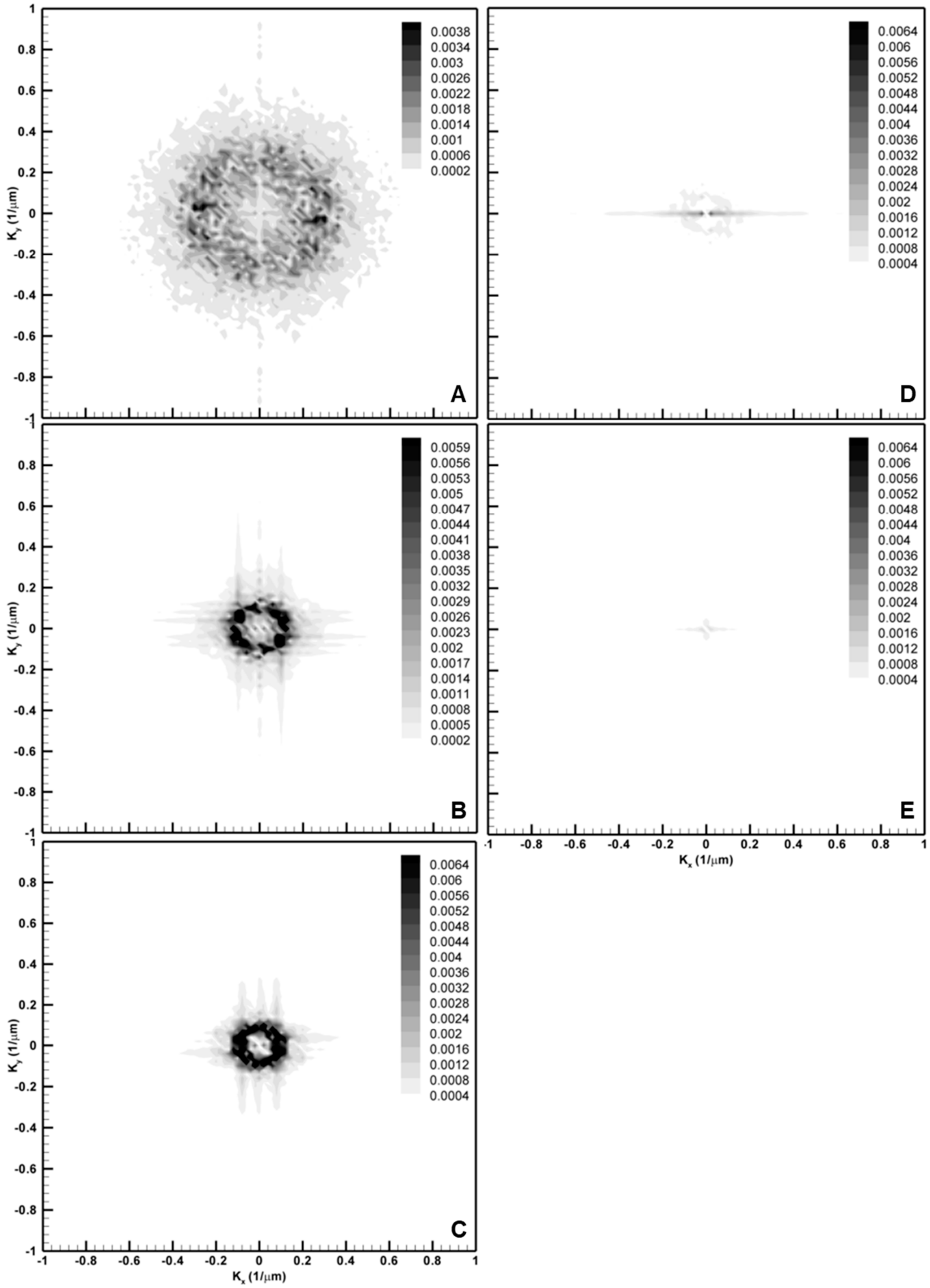

Figure S2. Spectra of WiMTiP samples with jammer thickness, t, of (**A**) 0, (**B**) 74, (**C**) 118 (**D**) 160um, (**E**) 340nm.

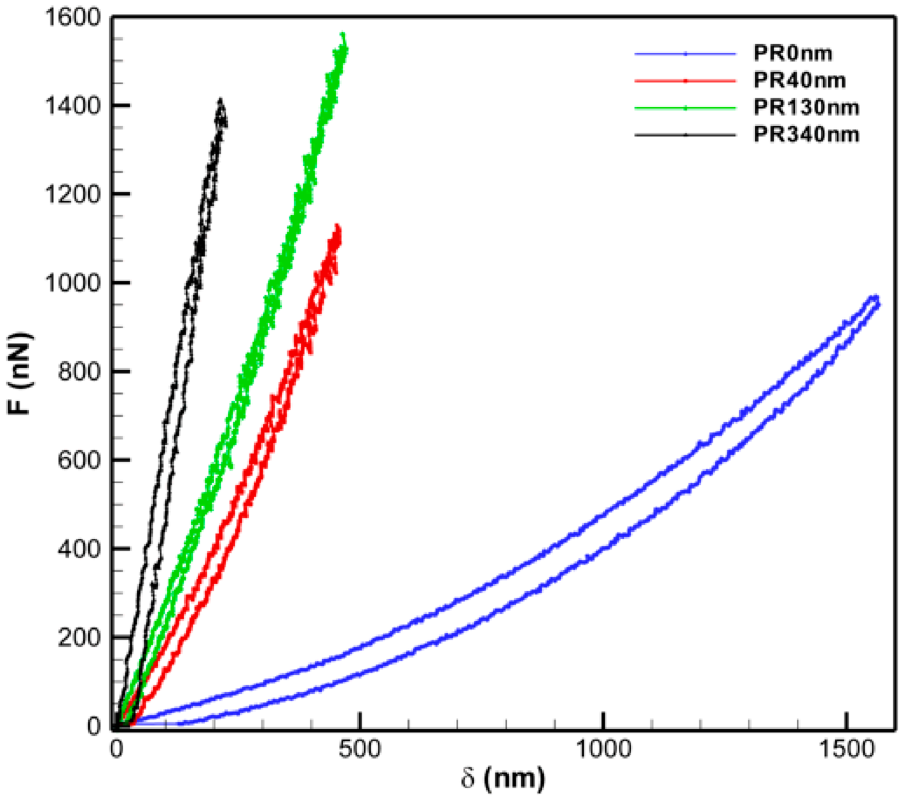

Figure S3. Sample $F - \delta$ curves of nano-indentation over an elastomer with a jammer layer of 0, 40, 130, 340nm thickness. Color coded by thickness.

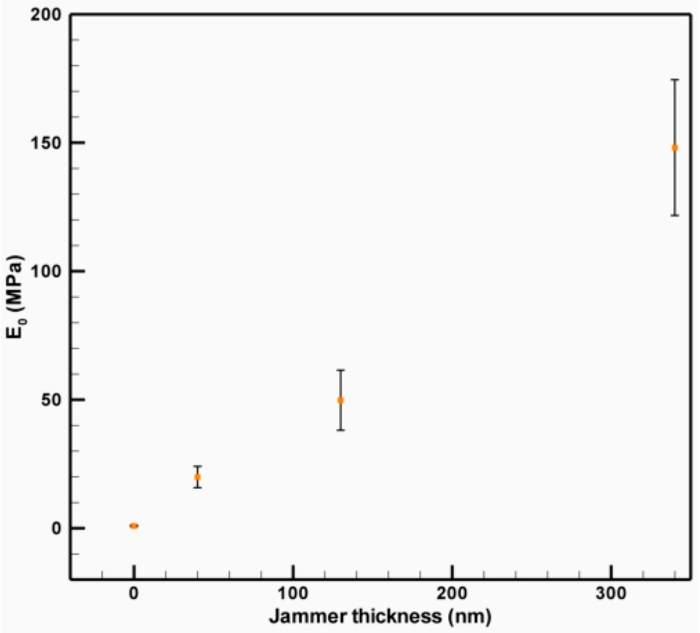

Figure S4. Hertian elasticity, E_0, vs the jammer thickness (nm).
